# Misuse of Over the Counter and Prescription Only Medication by Adults Accessing Specialist Treatment Services in the UK: A Narrative Synthesis

**DOI:** 10.1177/11782218221111833

**Published:** 2022-07-11

**Authors:** Rosalind Gittins, Louise Missen, Ian Maidment

**Affiliations:** 1 Clinical Department, Humankind, Durham, UK; 2 Aston Pharmacy School, Aston University, Birmingham, UK; 3 Gilead Sciences, London, UK

**Keywords:** Systematic review, over the counter, prescription only medication, misuse

## Abstract

**Background:**

Concerns about the misuse of over the counter (OTC) and prescription only medication (POM) are due to their impact upon physical/mental wellbeing, drug interactions and drug-related deaths. Improving an understanding of the pattern of use by people accessing specialist substance misuse services (SMSs) should enable improvements to treatment provision.

**Aim:**

To review the literature on the misuse of OTC/POM among adults accessing SMS, including the pattern of use, types of medication and associated characteristics.

**Methods:**

This review is reported in line with PRISMA. The protocol has been registered on PROSPERO (CRD42020135216) and separately published. A search of Cochrane, OVID Medline, Pubmed, Scopus and Web of Science databases and grey literature was undertaken. Only English language publications outlining OTC/POM misuse by adults in receipt of psychological/pharmacological interventions for substance misuse were included. Two reviewers conducted the title, abstract and full-text reviews using predetermined selection criteria and a piloted data extraction form to ensure a consistent approach. A third reviewer resolved disagreements and the Mixed Methods Appraisal Tool assessed for bias. Ethical approval was not required.

**Results:**

Thirteen studies with notable heterogeneity were included in the narrative synthesis after non-UK-based and ineligible publications were excluded, from the 143 potentially relevant papers. To reduce bias all studies were included in the analysis and GRADE-CERQual was applied. ‘High confidence’ was identified for all review findings, despite moderate methodological limitations. Antihistamine, benzodiazepine and opioid misuse was mentioned most frequently. Usage patterns and supply sources varied. Adverse consequences and polypharmacy are concerning. Withdrawal symptoms perpetuated misuse, often alongside illicit substance use, comorbid psychiatric/pain disorders and street drug shortages.

**Conclusion:**

OTC/POM misuse is common amongst adults accessing SMS. A renewed approach to withdrawal management is required. The limited number of studies may impact on generalisability but allowed for a more detailed review. Restricting to UK studies improved relevance due to drug market variations and availability of medicines in different countries. Further UK-based research on OTC/POM misuse in SMS is needed to build upon the current paucity in the published literature.

## Introduction

A variety of terms are used to describe when over the counter (OTC) and prescription only medication (POM) are used in a way other than as the manufacturers intended or as directed by a healthcare professional.^1,2^ In this research ‘misuse’ is used to describe the intentional inappropriate use of products (where the administration route or dose may be altered), for non-medical purposes.^3^ This term is also used to describe specialist treatment providers, since across the UK, they continue to be commissioned as ‘substance misuse services’ (SMSs). Although ‘misuse’ is contested by some who may view it as stigmatising and inaccurate, ‘*an exception may be claimed if people are using pharmaceuticals in ways that goes against advice from the supplier*’.^4^

OTC/POM misuse has been of growing concern for some time.^5,6^ Some of these medicines, (such as opioids and benzodiazepines) may be associated with dependency; they may also be used in combination with other medicinal products, illicit substances or alcohol.^7,8^ Polypharmacy is of particular issue where additive sedating/respiratory depressant effects and therefore drug-related death may occur, including concomitant use of alcohol, benzodiazepines, gabapentinoids and opioids.^9-12^ Indeed, drug related deaths in the UK remain at an all-time high and medicines continue to feature alongside illicit substances.^13,14^ Other adverse consequences can include negative effects upon physical and mental health, such as potentially fatal cardiac arrhythmias.^15-17^ Additionally, the socio-economic impact of misuse must not be underestimated and the person may be at increased risk of harm from themselves or to others.^18^

OTC/POMs can be obtained legally via prescriptions and pharmacy sales or illegally for example from street dealers or unregulated online sales.^19^ Misuse occurs for a variety of reasons, including for desired psychoactive effects or manage side-effects of recreational drug use, to self-medicate for withdrawal symptoms, psychiatric conditions and unmanaged pain disorders.^20-24^

Although the issue is thought to be increasing, due to an absence of a coordinated approach to national data monitoring regarding OTC/POM misuse, actual UK prevalence is unknown.^25^ Relevant data may be obtained using a multitude of different approaches, though each has their own strengths and limitations. Examples include clinical/dispensing/prescribing/sales management system searches, conducting wastewater analysis, drug tests, questionnaires, interviews, focus groups, netnography, pharmacovigilance and post-marketing surveillance.^26-38^ The most recent national review in England found that the issuing of antidepressants and gabapentinoids in primary care is increasing and benzodiazepine, opioid and z-drug prescribing remains prevalent, especially amongst older people and in areas with greater deprivation: other medication types and settings such as SMS were not considered and issues with dependency/withdrawal symptoms cannot be assumed from dispensing/prescribing data in isolation.^39,40^

It is important that effective interventions are promptly provided for OTC/POM misuse, given the association with increasing psychiatric issues, higher risk behaviours and reduced quality of life.^41,42^ Individuals’ characteristics may vary between countries due to drug market variations and availability of medicines, and differences may also exist when compared to people who primarily use illicit street drugs. For example, people who access UK SMS are usually men; however more medicines associated with dependency/withdrawal are being issued to women who may also be more at risk of misuse and adverse consequences including hospitalisations: the risk may further increase depending on the medication type, the persons’ age, ethnicity, HIV status, affluence and presence of chronic pain issues.^8,39,40,43-53^

Individuals who access SMSs have greater psychiatric comorbidity and associated medication use, and where OTC/POM misuse is disclosed, exhibit a greater severity of substance use and poorer mental health.^54-57^ Improved identification of affected individuals should improve SMS outcomes: currently there remains a paucity of evidence that supports OTC/POM misuse clinical management.^58^ Once engaged with SMS, people who misuse OTC/POM often respond well to therapeutic interventions, with high rates of successful treatment retention, completion and abstinence.^48,59,60^ It is therefore important that a review of the literature is undertaken to explore this further: this is thought to be the first review to consider the published literature on OTC/POM misuse by people accessing SMS.

The protocol for this review has been registered on PROSPERO (CRD42020135216) and separately published.^61^ It was undertaken, in line with Preferred Reporting Items for Systematic Reviews and Meta-Analyses (PRISMA) and using the PRISMA 2020 checklist.^62,63^

## Aim and Objectives

This review aims to examine the literature on the OTC/POM misuse by adults accessing SMSs. The objectives are to identify the types of medication being taken, the pattern of use and associated characteristics.

## Methods

As the protocol has been published^61^ a summary of the methods is reported here.

### Eligibility criteria

This review will consist of published studies which must meet all the following criteria:

- Adult participants (18 years or over)
- People who are misusing OTC/POM for non-medical purposes
- Individuals in receipt of psychological and/or pharmacological interventions from SMS for their substance use (in any setting eg, prison, community, inpatient) (excluding medication being prescribed for the management of their substance use)

There are no other restrictions on the publication type, population being studied or the date of publication. For practical reasons only English language results will be considered.

### Search strategy

In May 2021 an electronic search of Cochrane, OVID Medline, Pubmed, Scopus and Web of Science databases was undertaken.^64-68^ Additionally, a check of the grey literature and manual search of the reference lists to identify any further eligible studies ensured a comprehensive search. Truncation and a combination of keywords, medical subject heading and Boolean operator terms^69^ related to POM and OTC misuse in SMS such as (Over-the-Counter Drug Misuse) OR (Prescription Drug Misuse) AND (Substance Abuse Treatment Centres) was used. Aston University Library staff verified the suitability of the search strategy.

### Study selection and synthesis

Two independent reviewers (RG and LM) undertook the title, abstract and full-text reviews using predetermined selection criteria and a piloted data extraction form to ensure a consistent approach. Extracted data included publication author, year, title, journal name, study design, setting, methodology, sample size, statistical methods, OTC/POM medication details, use of other substances and summary of results and limitations. A third reviewer (IM) resolved any disagreements. Ethical approval was not required.

The Mixed Methods Appraisal Tool (MMAT) was used for all studies to consider the risk of bias as it accommodates all data types.^70^ Additional tools to assess the quality of the findings were planned to be utilised depending upon the publications identified, such as A Measurement Tool to Assess Systematic Reviews version 2 (AMSTAR2) for systematic reviews.^71^ Every study was given a qualitative summary and it was planned that if the data did not permit a meta-analysis, a Synthesis Without Meta-analysis (SWiM)^72^ would be undertaken. If feasible, it was planned that the I² test for heterogeneity would be implemented: heterogeneity would be considered likely if greater than 40% and in this instance it was planned that subgroup analyses would be undertaken.^73^ Cumulative strength of the findings were planned to be assessed using Grading of Recommendations, Assessment, Development and Evaluation (GRADE).^74^ Similarly, as for data extraction, the use of any supplementary, validated tools were applied by 2 independent reviewers (RG and LM) who discussed any disagreements with the third reviewer (IM) to further minimise bias.

## Results

### Search strategy findings

The search was undertaken on 10th May 2021 which provided a total of 3684 results from all databases included (see Supplemental File 1). This was reduced to 1989 after duplicates were removed. The grey literature was searched and all publications (total 2047) were checked by hand for potentially relevant references. Following this, publications were screened at title and abstract level, and ineligible publications excluded. This resulted in 143 potentially relevant papers being identified, so this was further restricted by including only UK-based publications which reduced the number to 13. The predominant reasons for exclusions were non-SMS, under 18 years of age or where the relevant data could not be extracted, such as mixed age ranges, in the case of Sakol et al.^75^

**Figure 1.**
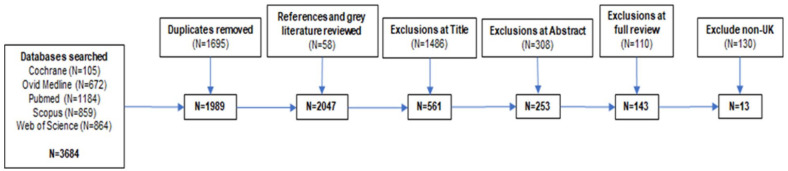
Summary of the publication selection process.

The 2 independent reviewers agreed in all instances: however, the third reviewer was consulted on one occasion, when determining the suitability to include Cooper^26^ since the online ‘treatment’ setting was notably different. The selection process is summarised in Figure 1 and the final papers selected for inclusion alongside a qualitative summary of their findings are stated in Table 1. As outlined in Table 2, studies using interviews and surveys/questionnaires wielded the greatest sample sizes: community/inpatient SMS settings were most prevalent.

**Table 1.** Summary of publications including key findings.

Study ID	Author(s)	Year	Title of publication	Journal name	Summary of findings
1	Armstrong et al	1992	The use of over-the-counter preparations by drug users attending an addiction treatment unit	*Br J Addict*	OTC misuse common. Easy to obtain in large amounts but not primary substance and only disclosed when asked. Duration of use and amount consumed varied greatly. Stimulants often used intermittently and regardless of street drug availability. Opioids often used for several days, for self-detoxification, if street drugs unavailable, to supplement use, for its own effect, to avoid withdrawal symptoms and experimentation.
2	Baird et al	2014	Gabapentinoid abuse in order to potentiate the effect of methadone: a survey among substance misusers	*Eur Addict Res*	Gabapentinoids used to potentiate methadone/to become intoxicated
3	Coombes et al	2019	Staff perceptions of prescription and over-the-counter drug dependence services in England: a qualitative study	*Addict Sci Clin Pract*	Staff concerns about stigma and lack of support, awareness, guidelines, pathways, funding and resources affecting treatment (especially for opioids). Current services perceived as inappropriate, variable and more suited for illicit drug users. Suggested service improvements include commissioning new services, developing national guidelines/pathways and increasing awareness.
4	Cooper et al	2013	‘I can’t be an addict. I am’. Over-the-counter medicine abuse: a qualitative study	*BMJ Open*	Mainly codeine combination product, but also decongestant and sedative antihistamines, usually started for genuine medical reasons. Considered themselves different from illicit users, self-blamed for losing control following cessation of prescribing. Subsequent use was for the ‘buzz’, obtained unproblematically via pharmacies/online. Withdrawal symptoms described, with work and health problems at higher doses. Mixed views about treatment options. Standard drug treatment services considered inappropriate. Concerns of ‘hidden addiction’ recorded in medical notes. Most supported continued OTC availability with addiction warnings and pharmacy training.
5	Fleming et al	1986	Dependence on dextromethorphan hydrobromide	*Br Med J*	Analysis of a sample of white powder believed to be an amphetamine of high purity detected dextromethorphan hydrobromide. Highlights need for continued vigilance as potential for abuse of any psychoactive drug and supports routine testing of substances (including drug checking).
6	Jaffe et al	2003	A postmarketing study of relative abuse liability of hypnotic sedative drugs	*Addiction*	Benzodiazepines have high abuse potential, commonly used, more than antidepressants and non-benzodiazepine hypnotics. More likely to be purchased to get high and on the street than via GP. Converse for antihistamines and other medicines used to aid sleep. Recommend benzodiazepines should not be prescribed with history of substance use: sedating antidepressants or non-benzo hypnotics could be alternatives.
7	McBride et al	1996	Three cases of nalbuphine hydrochloride dependence associated with anabolic steroid use	*Br J Sports Med*	Three case reports of nalbuphine hydrochloride dependence, obtained from illicit sources and alongside performance enhancing drugs. Supports further research into dependence potential of nalbuphine and relationship between anabolic steroid, other drug use and high-risk behaviours.
8	Oyefeso et al	1996	Prevalence and pattern of benzodiazepine abuse and dependence among patients in a methadone detoxification programme: a repeated cross-sectional analysis (benzodiazepine abuse among opiate addicts)	*Addiction Res*	Prevalence of benzodiazepine dependency, combined use of multiple benzodiazepines, cannabis, amphetamines and cocaine, obtained from GPs. Rates of injecting increased and age of first benzodiazepine use and prevalence of barbiturates decreased. On admission, for methadone detoxification need to routinely assess for use of benzodiazepines, barbiturates, cannabis and severity of dependence, and monitor treatment completion rates.
9	Perera et al	1987	The use of benzodiazepines among drug addicts	*Br J Addict*	High prevalence of benzodiazepines (especially diazepam). No difference in average age and sex ratios from non-users. Continued use for sleep, anxiety and withdrawal reactions. Minority stated using to intensify the ‘high’, to reduce or limit the quantity of their primary substance. Mostly oral, some injecting. Males more likely to use alcohol/greater polypharmacy. Majority cited easy (and initial) availability from GP/others with a prescription (parents/elderly relatives) as a reason for using.
10	Ruben et al	1992	Temazepam misuse in a group of injecting drug users	*Br J Addict*	Gel-filled temazepam capsules readily injected, causing medical complications. Obtained via GP, friends/relatives, street dealers, elderly people selling excess supplies, doctor shopping under false names/registering as a temporary resident, some attempted burglary. Used for desired drug effects, also sedating/relaxing effect to alleviate anxiety/depression. Some used as more available than heroin, to help sleep, commit crime or suppress opiate withdrawals. Some mixed with illicit heroin to provide a better ‘hit’. Temazepam tablets reported to be easier to inject than gel formulation.
11	Seivewright et al	1993	Withdrawal symptoms from high dose benzodiazepines in poly drug users	*Drug Alcohol Depend*	Withdrawal symptoms from high dose benzodiazepines prevalent amongst polydrug users, uncomplicated by simultaneous cessation of other drugs. Range of withdrawal symptoms similar but greater severity with higher doses, multiple benzodiazepines and oral use.
12	Strang et al	1994	Survey of use of injected benzodiazepines among drug users in Britain	*Br Med J*	Prevalence of benzodiazepine use, especially diazepam and temazepam. Notable levels of injecting, especially for temazepam capsules.
13	Thomas et al	2009	Diphenhydramine abuse and detoxification: a brief review and case report	*J Psychopharmacol*	Addiction to diphenhydramine significantly impacted on finances and travel to different community pharmacies. Withdrawal symptoms experienced within hours of missed doses. Experienced insomnia and memory impairment leading to accidents including fires, overdoses, blackouts and seizures. Highlighted need to ask about OTC/POM use and caution repeat requests.

**Table 2.** Summary of publication characteristics.

Study ID	Demographic characteristics of people misusing OTC/POM (eg, age, gender)	Sample size	Methodology
1	Range 20-49 years (mean 26.8 years)	53	Interviews
2	Not stated	129	Survey/questionnaire
3	*‘different genders, socioeconomic groups and ages. . .majority. . . middle-aged’*	15	Interviews
4	20-60 years, 48% male	25	Interviews
5	30 years, 100% male	1	Case report
6	78% male	297	Interviews
7	22-27 years, 100% male, 66.6% single and unemployed	3	Case report
8	Cohort 1: mean 28.7 years, 55% male, 55% single, 95% unemployed; Cohort 2: mean 27.7 years, 58.8% male, 43.1% single, 96.1% unemployed	71	Patient records
9	Mean 24.4 years, 64.6% male	79	Survey/questionnaire
10	19-26 years (mean 24.5 years), 74% male	23	Interviews
11	21-48 years (median 28 years), 54.5% male	33	Interviews and patient records
12	Mean 31 years, 67.8% male	208	Survey/questionnaire
13	56 years, 100% female	1	Case report

### Syntheses of study findings

Meta-analyses and subgroup analyses were not feasible and GRADE74 and AMSTAR271 were not indicated, due to an absence of randomised controlled trials and systematic reviews. Therefore a more structured analysis of heterogeneity using the I² test was not possible, though an initial review identified notable heterogeneity between publications. When considering the limited number of studies, their methodologies and (lack of) statistical findings, they were too dissimilar to be able to pool statistically and a narrative synthesis was conducted instead.^73,76^ It also proved difficult to implement the SWiM checklist due to the limited consistent information available and insufficient detail provided across the publications.^72^ Consequently, GRADE-CERQual (Confidence in Evidence from Reviews of Qualitative research)^77,78^ was applied to facilitate assessment of the confidence in the qualitative evidence synthesis, taking into account methodological limitations, coherence, data adequacy and relevance.

CERQual was utilised to add robustness to the qualitative narrative synthesis of the extracted data, which is summarised in Table 3. This approach considered the MMAT^70^ assessment that was undertaken by both reviewers, and determined the degree of confidence placed in the findings in relation to methodological limitations, coherence, data adequacy and relevance.^77,78^ Despite the methodological limitations being assessed as ‘moderate’ when the studies were considered as a whole, the CERQual assessment indicated overall ‘high confidence’ for each of the review findings: a similar approach has been exemplified by Lewin et al.^78^ Methodological limitations included lack of details provided in some studies, such as no mention of accounting for non-responder rates or confounding factors, or statistical/analysis methods were only partially reported or not disclosed at all.

**Table 3.** Summary of CERQual assessment.

Summary of review finding	IDs of studies contributing to the review finding	CERQual assessment of confidence	Explanation of CERQual assessment
Benzodiazepines, opioids and antihistamines are the most misused OTC/POMs by people accessing SMS	1, 2, 3, 4, 6, 7, 8, 9, 10, 11, 12, 13	High confidence	12 of the 13 studies specified at least one of these medicines (6 publications mentioned opioids, 9 mentioned benzodiazepines and 5 mentioned antihistamines)
When misused, OTC/POMs are usually taken orally or injected	4, 5, 7, 8, 9, 10, 11, 12, 13	High confidence	7 described oral, 6 described injecting (various) and 1 reported snorting, No other routes were reported/explicitly stated
OTC/POM misuse often occurs alongside illicit substance use, especially amphetamine, cannabis and opioids	1, 2, 3, 5, 6, 7, 8, 9, 10, 11, 12	High confidence	11 of the 13 studies specified illicit use and at least one of these substances was explicitly mentioned in 9 of them (6 mentioned amphetamines, 7 mentioned cannabis and 6 mentioned opioids). The remaining 3 studies did not provide any details (but an absence of details does not equate to no use)
The OTC/POMs being misused are sourced from a variety of places, including online, from (various) pharmacies/GPs, street dealers and friends/family	2, 3, 4, 5, 6, 7, 8, 9, 10, 11	High confidence	All stated at least one of these sources. The remaining 3 studies did not provide any explicit details
Adverse consequences are common and include complications from injecting, impact on personal finances, pharmacy bans, overdoses, accidents, criminal activity and physical health issues including problems with withdrawal and excessive paracetamol/ibuprofen from codeine combination products	1, 2, 4, 5, 7, 8, 10, 11, 13	High confidence	The remaining 4 studies did not provide any details about adverse effects (but an absence of details does not equate to no adverse effects being experienced). Withdrawal symptoms were a notable issue and contributed to perpetuating use
OTC/POMs are being misused for a variety of reasons, including to self-detox, for desired psychoactive effect, to experiment, to manage street drug shortages, psychiatric conditions, pain disorders, withdrawal symptoms, and to potentiate other substances	1, 2, 3, 4, 5, 6, 7, 9, 10, 11	High confidence	The remaining 3 studies did not provide any details about reasons for use. Withdrawal symptoms were a notable issue
There is significant variance in the pattern of OTC/POM misuse by people accessing SMS	1, 4, 7, 8, 9, 10, 11	High confidence	Significant variation in use reported on within and between publications, including duration, amount and frequency, ranging from single one-off/minimal use to routine daily heavy use. The remaining 6 studies did not provide any notable details about patterns of use.

To further reduce the risk of bias, and because every study met the inclusion criteria, every publication was included. Statistical methods were not routinely stated in all publications (such as case reports); therefore, no standardisation metrics or transformation methodologies were utilised.^72^

### Summary of findings

The following are the key findings from the narrative synthesis; further detail can be found in Table 3:

- *Benzodiazepines, opioids and antihistamines are the most misused OTC/POMs* by people accessing SMS. These medicines were mentioned in 12 of the 13 publications: in 9, 6 and 5 for benzodiazepines, opioids and antihistamines respectively. Three older studies mentioned products such as temazepam capsules which are no longer available in the UK.
- *When misused, OTC/POMs are usually taken orally or injected.* Seven studies described oral consumption (though not all stated this explicitly) and 6 described injecting (a mixture of intravenous, intramuscular and subcutaneous routes). One publication additionally mentioned snorting, and 4 studies did not clearly state the route of administration.
- *OTC/POM misuse often occurs alongside illicit substance use, especially amphetamine, cannabis and opioids.* Eleven of the thirteen studies specified the use of other substances and at least one of cannabis, amphetamine, and opioids was explicitly mentioned in 9 of them (in 7, 6 and 6 publications respectively). All stated substances were illicit, apart from alcohol, which was specified in only 4 publications. Two publications did not specify which substance was being used and 3 studies did not provide any details.
- *The OTC/POMs being misused are sourced from a variety of places*, including online, from (various) pharmacies/General Practitioners (GPs), street dealers and friends/family. The source was stated in 11 studies and at least one source was stated in all of them. The remaining 3 studies did not provide any explicit details about how the medication was obtained.
- *Adverse consequences are common* and include complications from injecting, impact on personal finances, pharmacy bans, overdoses, accidents, criminal activity and physical health issues, including problems with withdrawal and excessive inadvertent paracetamol (acetaminophen)/ibuprofen consumption from codeine combination products. Four studies did not provide any details about adverse effects. Withdrawal symptoms were a notable issue and contributed to perpetuating use.
- *OTC/POMs are being misused for a variety of reasons*, including to self-detox, for desired psychoactive effect, to experiment, to manage street drug shortages, psychiatric conditions, pain disorders, withdrawal symptoms, and to potentiate the effects of other substances. Similarly, to adverse consequences, withdrawal symptom management was frequently cited.
- *There is significant variance in the pattern of OTC/POM misuse* by people accessing SMS. Variation in use was observed both within and between publications, including in relation to duration, amount and frequency, ranging from single one-off or minimal use to routine daily heavy use. Where details were provided, in some cases the levels of use were significant and gave notable cause for concern.

Additionally, it was observed that the need for improvements to healthcare provision were frequently commented upon and 10 studies highlighted the need for increased awareness of OTC/POM misuse.

## Discussion

### Key findings

The prevalence of oral and injecting over any other routes of administration were notable and perhaps the latter is to be expected in the context of people who are actively injecting other substances. The variety of sources that OTC/POMs were obtained from have been identified by others.^19^ When considering adverse consequences and reasons for use, reference to withdrawal symptoms was notable across most of the studies. Adverse consequences, such as the impact on physical and mental health has been found by others.^15,17,18^

The frequent citing of benzodiazepines and opioids is in keeping with current national data trends: 6% of people accessing SMS report use of benzodiazepines and the 52% of people reporting opiates does not distinguish between traditional street drugs such as heroin, those available OTC or on prescription.^50^ Prevalent use of other substances, particularly opioids and cannabis were to be expected given that individuals were accessing specialist SMSs, where over half of people seeking support are known to have issues with opiates and almost one-fifth use cannabis.^50^

It was unexpected that alcohol or cocaine did not feature as frequently, since nearly half of individuals presenting to SMSs report problems with the former and 12% with the latter.^50^ Amphetamine was cited more commonly than would perhaps be expected as latest data suggests that only about 3% of people accessing SMSs currently report this substance as an issue.^50^ These differences may have been observed because older publications are unlikely to be reflective of current drug trends, or because the information wasn’t stated. It should be noted that some studies did not comment on concomitant use, though an absence of this information being provided does not equate to a lack of use.

The variance in the pattern of use (eg, from occasional to several times a day every day) is reflective of what is seen in SMS with other substances. Although some studies provided limited details, significant levels of use and exceptionally high doses were reported. This is of concern because of the associated risk of harms, especially when considering the degree of polypharmacy evidenced. Concomitant illicit drug use and the mention of substances which have additive sedating and respiratory depressant effects such as benzodiazepines and opioids is concerning because of their increased association with adverse consequences such as drug-related death due to accidental overdose.^9,10,12-14,79^

### Strengths and weaknesses

This is thought to be the first review of the published literature pertaining to OTC/POM misuse in SMS. Specialist librarian support from Aston University confirmed search strategy suitability, though significant manual searching was still required. However, this did allow for detailed checks of all reference lists to provide a level of assurance that the search was as thorough as possible. The study selection process is fully described and all publications are accounted for. The predominant use of qualitative methodologies demonstrates that their use to explore this subject area, in particular interviews, has been successful.

The final number of 13 studies meant that the subsequent analysis was limited and only tentative generalisations to the wider population accessing SMSs can be made. More (UK-only) papers could have been included if it had been possible to extract the data relating just to 18 years or over or to individuals recruited from SMSs rather than other services such as sexual health or homeless shelters. Restricting the publications to UK-only enabled more relevant findings as in other countries different medicines are available and drug markets also vary. Whilst this limited the number of papers and despite minimal resources, a more detailed assessment process was enabled. The inclusion of older papers led to the finding that 3 studies referenced products that are no longer available in the UK (particularly temazepam capsules) and it is also important to consider that patterns of misuse change over time: however, the medication is still available, albeit in different formulations and therefore they were still considered as being relevant for inclusion.^80^

Since all reviewers used the same data collection form, which was piloted for suitability of use, it enabled the data extraction process to be standardised. The heterogeneous nature of the extracted data, the lack of consistent data sets and small number of studies identified (as exemplified in Table 2), resulted in a narrative synthesis because of the inability to conduct meta-analyses or smaller sub-analyses. This led to the predetermined analysis and assessment tools not being applicable. Therefore, to consider the study limitations, the risk of bias and to provide an assessment of confidence in the review findings, the approach required reconsideration: the GRADE-CERQual criteria was applied in conjunction with the MMAT.^70,77,78^

CERQual identified a moderate limitation in the methodologies used, predominantly due to an absence of complete data sets and reporting of statistical analysis for some studies. Despite these limitations, as has been similarly described by Lewin et al^78^ a high confidence in all the review findings was found, which provides assurance, though the subjective nature of these tools must be considered. Having 2 reviewers independently undertaking study selection, data extraction, assessment of risk bias and review findings, with support from a third reviewer to resolve disagreements, strengthened the assurance process. Including all publications in the analysis allowed complete reporting of the dataset.

### Implications for clinicians, policymakers and researchers

The frequency that antihistamine misuse was mentioned is unexpected because they are not commonly observed in nationally reported trends; however an increase in their association with drug related deaths has been observed,^81^ suggesting the need for further exploration and increased vigilance for this amongst healthcare professionals. Although the level of detail provided about adverse consequences was often limited, this highlights an important need for SMS to consider approaches to the management of withdrawal symptoms and proactive interventions should be prioritised to reduce drug-related harms, especially relating to polypharmacy. Such an approach is similarly highlighted in the 2021 Department of Health and Social Care ‘Good for you, good for us, good for everybody’ report.^82^

The variety of sources used to obtain OTC/POMs highlights that making changes to supplies from pharmacies or prescribing changes in isolation (eg, in the case of codeine)^83^ would be unlikely to completely ameliorate availability, especially where online sales and street dealing remains ongoing: indeed such approaches may result in unintended consequences such as people seeking unregulated alternatives.^84^

The frequency that the need for improvement in raising awareness of OTC/POM misuse and other changes to healthcare provision was commented upon was notable. Despite the age of some of the publications, it was observed how much of an issue this remains to be, especially given the recent national review by Marsden et al.^39^ This highlights the need for continued developments and a greater understanding of how best to undertake these required improvements.

The heterogeneous and incomplete data set made it difficult to meaningfully identify themes for demographic characteristics. This, as well as the general limited number of studies identified and lack of prevalence data highlights the need for additional research to be conducted on this topic.

## Conclusion

Completion of this systematic review highlights the need for additional research to be conducted because of a paucity of the current published evidence base relating to the pattern of OTC/POM misuse by adults who are accessing SMSs. Benzodiazepines, opioids and antihistamines were most frequently cited as being misused, usually taken orally or by injection. Pattern of use varies significantly and they are sourced from a variety of places, including online, from pharmacies/GPs, street dealers and friends/family. Adverse consequences are common, as is the use of other substances, with amphetamine, cannabis and opioids being most prevalent. Withdrawal symptoms are a notable issue and contribute to perpetuating use. A limited number of relevant studies were identified which has consequent impact upon the strength of the analyses, associated findings and generalisability.

## Supplemental Material

sj-docx-1-sat-10.1177_11782218221111833 – Supplemental material for Misuse of Over the Counter and Prescription Only Medication by Adults Accessing Specialist Treatment Services in the UK: A Narrative Synthesis Click here for additional data file. Supplemental material, sj-docx-1-sat-10.1177_11782218221111833 for Misuse of Over the Counter and Prescription Only Medication by Adults Accessing Specialist Treatment Services in the UK: A Narrative Synthesis by Rosalind Gittins, Louise Missen and Ian Maidment in Substance Abuse: Research and Treatment
