# Supplementary material for: Misuse of Over the Counter and Prescription Only Medication by Adults Accessing Specialist Treatment Services in the UK: A Narrative Synthesis
Source: Subst Abuse. 2022 Jul 11;16:11782218221111833. doi: 10.1177/11782218221111833 (PMC9280808; doi:10.1177/11782218221111833)
Supplement: sj-docx-1-sat-10.1177_11782218221111833 – Supplemental material for Misuse of Over the Counter and Prescription Only Medication by Adults Accessing Specialist Treatment Services in the UK: A Narrative Synthesis [file sj-docx-1-sat-10.1177_11782218221111833.docx]

**Systematic Review Search Strategy**

*Undertaken 10th May 2021*

**Cochrane**

*Cochrane Reviews, Cochrane Protocols, Trials, Clinical Answers, Editorials and Special Collections*

| Search Number | Search Term | Number of Results |
| --- | --- | --- |
| #1 | "POM" OR "prescription medic*" OR "over the counter" OR "OTC" OR "pharmacy medic*" OR "pharmacy only medic*" (Word variations) | 3892 |
| #2 | MeSH descriptor: [Prescription Drugs] explode all trees | 108 |
| #3 | MeSH descriptor: [Nonprescription Drugs] explode all trees | 188 |
| #4 | #1 OR #2 OR #3 | 2426 |
| #5 | (misus* OR abus*) | 17706 |
| #6 | (treat* OR service*) | 917259 |
| #7 | #4 AND #5 AND #6 | 105 |

**Ovid Medline**

*Ovid MEDLINE(R) and Epub Ahead of Print, In-Process, In-Data-Review & Other Non-Indexed Citations, Daily and Versions(R) 1946 to 7/5/2021*

| Search Number | Search Term | Number of Results |
| --- | --- | --- |
| #1 | ("POM" OR "prescription medic*" OR "over the counter" OR "OTC" OR "pharmacy medic*" OR "pharmacy only medic*").tw | 20491 |
| #2 | Prescription Drugs/ | 6290 |
| #3 | exp Nonprescription Drugs/ | 6269 |
| #4 | 1 or 2 or 3 | 28905 |
| #5 | (misus* or abus*).tw. | 158368 |
| #6 | (treat* or service*).tw. | 6220478 |
| #7 | 4 and 5 and 6 | 672 |

**Pubmed**

| Search Number | Search Term | Number of Results |
| --- | --- | --- |
| #1 | "POM" OR "prescription medic*" OR "over the counter" OR "OTC" OR "pharmacy medic*" (Text Word) | 20,188 |
| #2 | “Prescription Drugs” OR “Nonprescription Drugs” (MeSH Terms) | 16,048 |
| #3 | #1 OR #2 | 32,091 |
| #4 | misus* OR abus* (Text Word) | 213,469 |
| #5 | treat* OR service* (Text Word) | 7,122,263 |
| #6 | #3 AND #4 AND #5 | 1,184 |

*((("POM"[Text Word] OR "prescription medic*"[Text Word] OR "over the counter"[Text Word] OR "OTC"[Text Word] OR "pharmacy medic*"[Text Word]) OR ("Prescription Drugs" OR "Nonprescription Drugs"[MeSH Terms])) AND (misus*[Text Word] OR abus*[Text Word])) AND (treat*[Text Word] OR service*[Text Word])*

**Scopus**

| Search Number | Search Term | Number of Results |
| --- | --- | --- |
| #1 | "POM" OR "prescription medic*" OR "over the counter" OR "OTC" OR "pharmacy medic*" OR "pharmacy only medic*" (All Fields) | 132,459 |
| #2 | misus* OR abus* (All Fields) | 1,148,726 |
| #3 | treat* OR service* (All Fields) | 24,476,019 |
| #4 | #1 AND #2 AND #3 | 10,410 |
| #5 | #4, Restrict to Article Title/Abstract/Keywords | 9,776 |
| #6 | #5, Restrict to Title/Abstract/Keywords | 859 |

*TITLE-ABS-KEY ( ( treat* OR service* ) AND ( misus* OR abus* ) AND ( "POM" OR "prescription medic*" OR "over the counter" OR "OTC" OR "pharmacy medic*" OR "pharmacy only medic*" ) )*

**WoS**

| Search Number | Search Term | Number of Results |
| --- | --- | --- |
| #1 | ALL FIELDS: "POM" OR "prescription medic*" OR "over the counter" OR "OTC" OR "pharmacy medic*" OR "pharmacy only medic*" (Core Collection) | 33,240 |
| #2 | ALL FIELDS: misus* OR abus* (Core Collection) | 400,269 |
| #3 | ALL FIELDS: treat* OR service* (Core Collection) | 10,310,975 |
| #4 | #1 AND #2 AND #3 | 864 |

*((misus* OR abus*) AND (treat* OR service*) AND ("POM" OR "prescription medic*" OR "over the counter" OR "OTC" OR "pharmacy medic*" OR "pharmacy only medic*"))*
